# Public Reaction towards the Potential Side Effects of a COVID-19 Vaccine: An Italian Cross-Sectional Study

**DOI:** 10.3390/vaccines10030429

**Published:** 2022-03-11

**Authors:** Serena Barello, Lorenzo Palamenghi, Guendalina Graffigna

**Affiliations:** 1 EngageMinds HUB—Consumer, Food & Health Engagement Research Center, Università Cattolica del Sacro Cuore, 20123 Milan, Italy; serena.barello@unicatt.it (S.B.); guendalina.graffigna@unicatt.it (G.G.); 2 Department of Psychology, Università Cattolica del Sacro Cuore, 20123 Milan, Italy; 3 Faculty of Psychology, Università Cattolica del Sacro Cuore, 20123 Milan, Italy; 4 Faculty of Agriculture, Food and Environmental Sciences, Università Cattolica del Sacro Cuore, 26100 Cremona, Italy

**Keywords:** COVID-19, vaccine hesitancy, public health, public health communication

## Abstract

In March 2021, the possible link between the Oxford–AstraZeneca vaccine and some cases of blood clots lead several governments to suspend the administration of said vaccine, or to adjust their administration strategies, regardless of the fact that both EMA and WHO claimed the benefits of the vaccine to far outweigh its risks. The lack of a coordinated decision-making process between different health authorities possibly had an impact on people’s trust in the health authorities themselves, and on their willingness to vaccinate against COVID-19. In this study, we assessed the impact of the Astrazeneca case on a sample of 1000 Italian participants. The results demonstrate that a large part of the population is willing to delay the vaccination to be granted a vaccine perceived as “better”. We also assessed the importance of several socio-demographic and psychological factors in predicting hesitancy and discuss the implications for public communication strategies.

## 1. Introduction

The effectiveness of an anti-COVID-19 vaccine does not only depend on the effectiveness of the vaccine itself, but it is also linked to the number of citizens who adhere to the vaccination campaign. Many studies across the world demonstrate that exposure to negative misinformation about COVID-19 vaccines significantly reduces public intention to be vaccinated, while also increasing skepticism towards immunization campaigns [1,2,3]. According to an increasing number of studies, due to the “hesitancy” phenomenon, even if an effective and safe vaccine is indeed available, a substantial proportion of citizens may refuse or delay the vaccination with a notable number of studies reporting COVID-19 acceptance rates below 60%, which would pose a serious issue for efforts to control the COVID-19 pandemic [4].

Moreover, lessons learned from the H1N1 2009 pandemic flu showed that public engagement with immunization programs requires considerable efforts to increase trust in healthcare authorities, plus public campaigns capable of listening to citizens’ worries, and to address their doubts [5]. Previous research indicates that citizens’ adherence to vaccine behaviors is variable and inconsistent; thus, a successful vaccination plan will necessarily require widespread public campaigns to sensitize citizens about the importance of getting vaccinated to control the pandemic spread.

**Figure 1:**
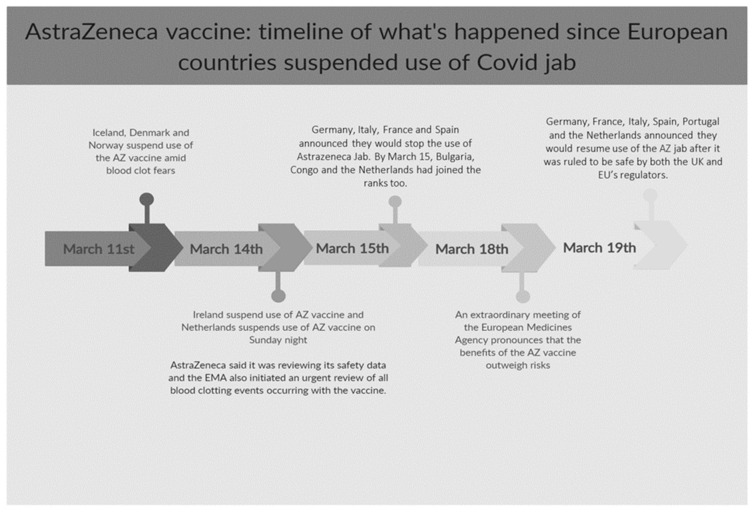
AstraZeneca vaccine: timeline of what happened since countries worldwide suspended the use of the AZ vaccine.

However, on the evening of 15 March 2020, a possible connection between the AZ vaccine and rare blood clots has pushed international governmental agencies to revise recommendations on who should receive this specific vaccine. This fact made several EU countries (among which France, Germany, and Italy) take the decision to suspend the provision of the AZ vaccine and/or to change the target of the vaccine based on potential health risks, regardless of the fact that no causality assessment was indeed available at that time. Figure 1 presents a timeline of events ending on Friday 19 March when newspapers reported the restart of AZ administration in many European Countries. In Italy, the National Medicines Agency (AIFA) interrupted the provision of the AZ batch ABV2856 on 11 March. Four days later, the AIFA suspended all AZ vaccinations until 18 March, while allowing the administration of the Comirnaty vaccine to continue. Indeed, this event would potentially threaten the success of COVID-19 vaccination campaigns, which is crucial in the management of the COVID-19 pandemic.

A recent publication indeed shows that the Italian citizens’ reaction to the media coverage of the (presumed) association between the blood clots and the Astrazeneca vaccine resulted in about 10–20% vaccine candidates refusing vaccination with this vaccine, thus delaying the vaccination and causing about 200,000 doses not being administered [6].

Misinformation regarding the COVID-19 pandemic is a serious threat to public health, ranging from the proliferation of damaging health advice, such as ingesting bleach, to politically motivated conspiracies about the origin of the virus. The proliferation of misleading information about COVID-19, how it spreads, how to treat it, and who is ‘behind’ it, has prompted the World Health Organization to warn about the risk of an ‘infodemic’ [7]. However, the scope of public health communication related to vaccination is wide and it is complex to maintain in time a full coherence and consistence of the messages sent to the population about the importance of adhering to preventive measures. Indeed, governmental communication and decisions may impact citizens’ risk perception and trust in the health authorities [8].

Regarding the case of the AZ vaccine, it is plausible that the media debate about its supposed side effects might have shaped peoples’ psychological beliefs and fostered vaccine hesitancy. Although tragic, this unfortunate communicative event offers to the social sciences a “real world case” to assess the impact of uncertain public health messages on vaccine hesitancy and citizens’ attitudes towards vaccination during a pandemic. A better understanding of the psychology of hesitant individuals after the AZ vaccine communication events might offer a more complete understanding of why these individuals consider a COVID-19 vaccine in a positive or negative way. By identifying these distinguishing characteristics, health agencies may be better able to identify who in the population is more likely to be hesitant about a COVID-19 vaccine.

Scholars are increasingly recognizing that, to comprehend the phenomenon of vaccine hesitancy, insights from the social and behavioral sciences play an important role, especially regarding the spread of misinformation about the virus [9,10,11]. Most studies focusing on the determinants of vaccine hesitancy report sociodemographic variables as significant predictors of influenza vaccine hesitancy. For example, a number of studies reported an association between the ethnicity of a study population and influenza vaccine uptake [12,13,14,15,16]. Possible explanations for these results might be differences in access to health care services [17], stigma and discrimination from care providers [18], and negative attitudes towards vaccination [19], among others. While such variables may be significantly related to vaccine hesitancy or their predictors, they cannot be used as stand-alone factors to explain its emergence or intensity in individuals. On the other side, literature suggests that addressing vaccine hesitancy involves developing a deep understanding of the psychosocial and attitudinal dimensions behind vaccine acceptance [20]. Psychologists have long demonstrated that people, when requested to enact health or preventive behaviors, are deeply influenced by their beliefs and attitudes towards the behavior itself, and this happens also in the context of vaccination [21,22].

To date, many psychological variables have been explored in relation to vaccine hesitancy. For example, altruistic beliefs [23], the personality traits neuroticism and conscientiousness [24], locus of control [25], and cognitive style [26], have each been shown to affect vaccine hesitancy. Vaccine hesitance has also been associated with conspiratorial, religious, and paranoid beliefs [27], while mistrust of authoritative members of society [3,13], such as government officials, scientists, and health care professionals, has been linked to negative attitudes towards vaccination [5,28,29,30,31] and to low level of individuals’ health engagement [32]. Taken together, the existing literature indicates that there are likely to be several psychological dispositions that include personality, cognitive styles, emotion, beliefs, trust, and socio-political believes and attitudes that distinguish those who are hesitant or resistant to a COVID-19 vaccine from those who are accepting.

In the present study, we present the results from a survey about vaccine hesitancy and related determinants on a representative sample of Italian adult individuals in the days in which media-diffused news about the presumed side effects of the AZ vaccine and the Italian health authorities decided to suspend this vaccine. The study aimed at disentangling not only the impact of such events on the rate of vaccine hesitant persons in Italy, but also to understand what socio-demographic and psycho-social factors appeared more predictive of individuals’ vulnerability to these events.

*Objective 1*. First, we sought to measure the impact that the media coverage of the alleged blood clots associated with the AZ vaccine exerted on the willingness of the Italian adult population to delay the vaccination, hoping for a perceived “better” vaccine in the future.

*Objective 2.* Second, we tried to identify the socio-demographic characteristics that make individuals more prone towards delaying the COVID-19 vaccination.

*Objective 3.* Third, we sought to identify the most salient psychological characteristics that distinguish individuals who are intentioned to delay the COVID-19 vaccine from those who accept it more promptly.

## 2. Materials and Methods

### 2.1. Participants and Procedure

1002 Italian citizens aged between 18 and 70 years old were recruited by a professional panel provider (Norstat Italia s.r.l., Milano, Italy). After providing their informed consent, participants were asked to fill an online survey (CAWI-computer assisted web interview-methodology). Data were then weighted to reach the desired quotas, representative of the Italian adult population in that age range. Assigning sample weights to participants is a common strategy to compensate for distortion in sampling due to self-selection and non-response biases [33]; in our case, sample weights were calculated by the panel provider. Participants who did not answer the question regarding their family’s average wage (136 weighted cases), were removed listwise; hence, the sample was composed of 866 cases.

Data collection occurred between 12 March 2021 and 17 March 2021, hence during the days of the “AstraZeneca” case.

Each participant was instructed about the aims of the research and gave informed consent before starting the questionnaire. By agreeing to start the compilation, participants accepted the informed consent. They were also allowed to drop out from the compilation at any time. The GDPR compliance for the participants here involved was guaranteed by Norstat s.r.l. We received the anonymous database for analysis. No participants’ identification detail was provided to researchers.

### 2.2. Measures

Participants were asked a series of questions regarding their socio-demographic profile. Participants were profiled according to:

- Gender;
- Age;
- Italian region of residence, which was then recoded to divide the sample into four main geographical areas (north-west, north-east, center, and south and islands);
- Education: participants were asked their higher-ranked school degree, and were then grouped into three clusters (middle school or lower, high school degree, university degree or higher);
- Average family net income on a monthly basis, participants were then divided in two groups (above and below the median value of 1800 €/month);
- Previous vaccinal behavior (“have you ever been vaccinated against influenza?”) answered in a yes/no fashion;
- Closeness to COVID-19: participants were asked three questions (“have you ever been diagnosed with COVID-19, confirmed by a positive test?”, “has one of your relatives been diagnosed with COVID-19, confirmed by a positive test?”, and “have you been quarantined due to a close contact with a suspect COVID-19 case?”) and were asked to answer yes/no to each one.

Additionally, participants were asked to fill a brief psychological survey regarding their attitude towards vaccines in general, their conspiracy mentality, and their perception of the threat posed by COVID-19 in various contexts. In particular, beliefs in conspiracy theories were assessed using the CMQ questionnaire [34], a brief scale composed of 5 statements which participants need to rate according to how much they perceive those to be true (from 0% to 100%). Attitude towards vaccines was measured using the 5C scale [35], a measure composed of 15 statements that participants answered using a 7-points agreement scale; the scale was developed starting from a theoretical model that describes 5 different psychological antecedents of vaccination (i.e., confidence, complacency, constraints, calculation, and collective responsibility). Finally, the perceived risk was measured by a series of 12 ad hoc items describing a series of activities (e.g., going to the restaurant, getting on a bus, etc.), for which participants were asked to rate how they feel that specific activity to be risky regarding COVID-19 on a scale going from 1 (very little risky) to 5 (very risky).

Finally, participants’ willingness to delay the vaccination against COVID-19 was measured by asking them to rate on a 5-point scale the agreement to a single statement (“I am willing to wait in order to receive the vaccine that I deem the best”). Participants were then divided into two groups: willing to delay (i.e., participants who answered 4, “I agree a lot”, or 5, “I absolutely agree”) and participants not willing to delay or neutral.

### 2.3. Data Analysis

All analyses were carried out using IBM SPSS v27 (Release 27.0.0.0).

Prior to computing summatory scores for the scales, reliability was assessed using Cronbach’s α; a value above the 0.75 threshold is generally considered acceptable. Summatory scores were computed by averaging the items of each scale (after reverse-coding items where necessary). Given the variety in values’ ranges, summatory scores and age were standardized in z-scores to allow better comparability.

A logistic regression was then carried out to assess whether socio-demographic characteristics and psychological variables can help predicting the probability of a person being intentioned to delay the vaccination over the probability of not being intentioned to. Forward Wald method was used to only include variables statistically significant at 5%. Variables were divided in two blocks: a first block composed of socio-demographic variables, and a second block with psychological variables (as listed above).

## 3. Results

The sociodemographic characteristics of the included sample and the distribution of the categorical variables included in the model are reported in Table 1. Table 2 shows the descriptive statistics of continuous variables and, where applicable, the reliability index (Cronbach’s α) of composite scores.

Data showed that almost half of our sample (46%) agreed with the statement that they were willing, if given the chance, to delay the vaccination to wait for a “better” vaccine. All the used scales showed an acceptable reliability (Cronbach’s α > 0.75).

A first logistic regression model was run to assess whether socio-demographic variables (gender, age, geographical area, level of education, average wage, previous flu-shots, and being found positive/a family member being found positive to COVID-19 and/or having been quarantined due to contact with a positive person) can help predicting the intention to delay the vaccination against COVID-19 to wait for a “better” vaccine. Continuous variables were standardized by transforming them in z-score. The model resulted statistically significant [χ^2^_(3)_ = 16.052, *p* = 0.001], although not particularly predictive (Nagelkerke’s R^2^ = 0.025, 57% of correctly classified subjects in the confusion matrix). Results demonstrate that, among the variables included in our analyses, only education level and previous vaccinal behavior are significant. In particular, our first model showed that those who vaccinated against influence in the past are about 32% less likely to be intentioned to delay the COVID-19 vaccine (*p* = 0.010); moreover, people with a high school degree resulted, instead, about 60% more likely to be intentioned to delay the vaccination than people without a higher degree (*p* = 0.016).

A second block was added to the logistic model, including psychological constructs (i.e., risk perception of COVID-19, conspiracy beliefs, and the 5C precursors of vaccine intention).

The omnibus test shows that the model resulted statistically significant [χ^2^_(6)_ = 116.644, *p* < 0.001] and that this block significantly improved over the previous model [χ^2^_(3)_ = 100.592, *p* < 0.001]. The model’s predictive power was also improved (Nagelkerke’s R^2^ = 0.168; 67.5% of correctly classified subjects in the confusion matrix).

Overall, the model shows that risk perception, conspiracy beliefs, and the “calculation” factor of the 5C precursors (i.e., the reliance on a “rational” calculation of perceived benefits vs. risk implied by the vaccination) are a significant predictor of the intention to delay the vaccination. In particular, participants with a higher perceived risk of COVID-19 showed a higher likelihood of being hesitant (+48% for each standard deviation above the average, *p* < 0.001); participants more oriented towards conspiracy mentality have a higher probability to have the intention to delay the vaccination (+53% for each standard deviation above the average, *p* < 0.001), as well as participants that reported a higher propensity to rely on rational calculations for deciding regarding vaccinations (+42% for each standard deviation above the average, *p* < 0.001).

Table 3 shows the variables included in the model (after block 2 was included) and their respective odds ratios.

## 4. Discussion

Within the public arena about COVID-19 vaccines, a central place was occupied by the AstraZeneca vaccine, as its efficacy/safety balance has been an object of discussion not only in the scientific arena, but also in the public sphere. The media coverage about the presumed side effects of the AZ vaccine in early March 2021 in Italy, together with some changes in governmental decisions about the ongoing vaccination campaign, fostered citizens’ hesitancy about the vaccine. This suspension offers a “real world” setting for understanding the public motivation behind vaccination intention (and, on the contrary, reasons for vaccine hesitancy) after safety concerns over the Oxford-AstraZeneca vaccine.

In this study, we collected public reactions towards the possible severe side effects of a specific vaccine against COVID-19, and we also analyzed the psychological factors that contribute to the intention to delay the vaccination behavior, regardless of the fact that the vaccine for which the possible side effects were reported by the media (namely, the Oxford–AstraZeneca vaccine) was demonstrated to be safe and effective in adults aged 18 years and older [36]. Still, national health agencies have moved ahead with their own risk and benefit assessments, resulting in very dissimilar conclusions: from limiting the vaccine’s use to specific age groups to suspending its usage. Because of this lack of coherence in decision making, scholars and experts warn, public trust towards science and healthcare agencies dramatically decreased [37].

In this light, our findings are concerning because the public response to the news of possible severe side effects of a COVID-19 vaccine seems to have further fostered vaccine hesitancy, potentially slowing down the campaign, as other studies seem to imply [6,38].

Previous studies have reported and argued that variables, such as gender, age, and other socio-demographic characteristics are important explanatory factors of vaccination hesitancy [39,40,41].

However, in our study, socio-demographic variables do not seem to be an important predictor. Indeed, it is important to note that most sociodemographic variables in our study make little contribution to explain vaccine hesitancy on an individual level; this in line with previous studies [42], which reached inconclusive, or contradictory, findings in the section of sociodemographic variables.

Indeed, sociodemographic variables are, at best, a way to identify groups of (more or less) homogeneous people with similar levels of hesitancy, or to identify groups more at risk of non-adherence; however, belonging to a specific social cluster (defined e.g., by gender, age, education, and wage) cannot be, from a psychological perspective, a sufficient explanation for a specific behavior, without further investigation. Accordingly, in our study psycho-attitudinal factors appeared to have a more important role in determining vaccine hesitancy. Indeed, in our study, the most relevant predictors of hesitancy were psychological, namely: conspiracy beliefs, the calculation of risk/benefits, and the perceived risk of COVID-19. In particular, as expected, our results demonstrated that people with a tendency to have conspiracy beliefs are generally more prone to be hesitant. More surprisingly, our results demonstrated that people who feel more at risk due to COVID-19 are more likely to be willing to wait for a “better” vaccine. While this feels contradictory, our interpretation is that there might be some underlying personality traits [43], which might lead people to be both more worried for the consequences of COVID-19 and, at the same time, more cautious towards a solution that might pose some health risks; this is also coherent with the fact that in our study people that claimed to rely more on a rational calculation of risks and benefits for their decision to be vaccinated, were generally more prone to hesitate and decide to wait for a “better vaccine”. However, our data are not fit to test this hypothesis, which will have to be tested and corroborated in future studies.

While our study did not directly address the question of how to effectively shape a public health campaign in order to reduce vaccine hesitancy, our results have some interesting implications toward this regard: indeed, understanding the psychological roots of citizens’ vaccine hesitancy has substantial implications on the possible development of a public health communication campaign to promote the national vaccination plan [44,45]. Public education programs aimed to sustain citizens’ literacy about the vaccines can be helpful to some extent, but not enough [3,46]; the explicative power of the “calculation” factor in determining vaccine hesitancy corroborates the fact that it is not only a matter of quantity of information about vaccination that the target should receive, but also of the way such information are processed, interpreted, and even distorted by individual’s psychology. Similarly, the tendency to conspiratorial beliefs is another element that characterizes high levels of vaccine hesitancy after a communication crisis and cannot only be managed by emphasizing the diffusion of scientific information about the safety and effectiveness of the vaccines. These data, indeed, demonstrate how identifying the inner motivational and attitudinal factors for vaccination hesitancy and then proactively tailoring public health messaging and incentives to address these factors prior and along an immunization program may improve overall vaccine uptake [7].

As a final remark, this study has some limitations: first and foremost, the study assesses the intention to delay the vaccination, but no data were generated regarding actual behaviors. Second, in order to effectively measure the impact of the news regarding the supposed link between the AZ vaccine and rare blood clots, a pre-post study would have been necessary. Indeed, our study aimed at identifying the public reaction to the news regarding AZ, the non-coherent decision making by diverse health authorities, and the characteristics that made the intention to delay the vaccination more likely. Finally, the question used as the dependent variable generically referred to “a better vaccine”, while not stating explicitly what “better” implied. This was done in order to allow our participants to freely interpret what “better” means, and answer accordingly; nevertheless, given the framing of the questionnaire and of the media debate in that period, it is much more likely that most participants referred to a “better” vaccine in terms of safety (i.e., the presence of severe side effects).

## Figures and Tables

**Table 1:** Sample characteristics.

Variable	N	%
Gender		
Female	426	50.8
Male	440	49.2
Geographical area		
N-W	226	26.1
N-E	151	17.5
Center	174	20.1
South and islands	314	36.3
Education		
Middle school or lower	144	16.6
High school	503	58.1
University or higher	219	25.3
Family income		
Below median (≤1800 €/month)	475	54.9
Above median (>1800 €/month)	391	45.1
Previously vaccinated for influence		
Yes	293	33.8
No	573	66.2
Confirmed (by test) COVID-19 diagnosis in the past		
Yes	109	12.5
No	757	87.5
Confirmed (by test) COVID-19 diagnosis for a relative in the past		
Yes	204	23.6
No	661	76.4
Quarantined due to close contact with a suspect COVID-19 case		
Yes	181	21
No	684	79
I am willing to wait to receive a vaccine which I think is better		
Agree	398	46
Disagree/neutral	467	54

**Table 2:** Descriptive statistics and reliability indices.

Variable	Mean (std. dev.)	Min–Max	Skewness	Kurtosis	Cronbach’s α
Age	46 (13)	18–70	−0.07	−0.93	N/A
Risk perception	3.79 (0.72)	1–5	−0.80	1.19	0.92
Conspiracy beliefs	66.15 (20.07)	0–100	−0.49	0.05	0.89
Confidence	5.00 (1.50)	1–7	−0.80	0.13	0.89
Complacency	3.30 (1.59)	1–7	0.22	−0.84	0.80
Constrains	2.85 (1.63)	1–7	0.43	−0.86	0.85
Calculation	5.04 (1.39)	1–7	−0.62	0.01	0.82
Collective responsibility	5.38 (1.39)	1–7	−0.56	−0.22	0.76

**Table 3:** Results from the logistic model after the second block was added.

Variable	B	S.E.	Wald	*p*-Value	Odds Ratio *
Education			7.478	0.024	
Education (high School)	0.559	0.206	7.348	0.007	1.749
Education (university)	0.384	0.237	2.616	0.106	1.468
Previously vaccinated	−0.435	0.158	7.586	0.006	0.647
Risk perception (z score)	0.390	0.080	24.064	<0.001	1.477
Conspiracy mentality (z score)	0.426	0.078	29.529	<0.001	1.531
Calculation (z score)	0.351	0.078	20.123	<0.001	1.420

* Ratio of the probability of being intentioned to delay the vaccination over the probability of not being intentioned to delay.

## Data Availability

Data are available upon reasonable request to the corresponding author.
